# A retrospective study on the effectiveness and safety of the combination of intranasal corticosteroid and intranasal antihistamine in the management of patients with allergic rhinitis

**DOI:** 10.3389/falgy.2026.1863171

**Published:** 2026-07-22

**Authors:** Anil Kumar Monga, Tanmay Rane, Monika Chinda, Sagar Bhagat, Saiprasad Patil, Hanmant Barkate

**Affiliations:** 1 Uday ENT Hospital, New Delhi, India; 2 Glenmark Pharmaceuticals Ltd., Mumbai, India

**Keywords:** allergic rhinitis, antihistamine, azelastine hydrochloride, corticosteroid, INAH, INCS, intranasal spray, mometasone furoate

## Abstract

**Background:**

The Allergic Rhinitis and Its Impact on Asthma 2025 guideline recommends the combination of intranasal corticosteroid and intranasal antihistamine to be used as first-line treatment for moderate-to-severe allergic rhinitis because of its superior symptom control. This study was conducted to examine real-world evidence regarding the effectiveness and safety of the combination of Mometasone furoate and Azelastine (MF–Az) nasal sprays in Indian patients with allergic rhinitis.

**Methods:**

This multicentric, retrospective study was carried out across 424 ENT clinics in India. Medical records were scrutinized for information such as medical history, symptoms, clinical results, and adverse events (AEs). The mean change in total nasal symptom scores (TNSS) and total non-nasal symptom scores (TNNSS) from baseline to end of treatment was used to assess effectiveness. Independent EC approval was obtained before initiating the study.

**Results:**

The medical records of 4,500 patients with allergic rhinitis from 424 ENT clinics across India were screened. The mean change from baseline TNSS and TNNSS at Day 7 were −1.97 (±2.58) and −1.55 (±2.63), respectively, while those at Day 14 were −3.62 (±3.99) and −2.70 (±3.84), respectively. These changes were statistically significant (*p* < 0.0001). There was no hospitalization, serious AEs, or treatment discontinuation due to AEs among patients.

**Conclusion:**

The study concluded that intranasal MF–Az spray significantly alleviated nasal and non-nasal symptoms and was well tolerated.

## Introduction

Allergic rhinitis (AR) is a hypersensitivity disorder driven by IgE-mediated immune mechanisms involving Th2 inflammatory pathways. AR has been reported to affect one in six individuals and is associated with significant morbidity, loss of productivity, and costs related to healthcare (1–4).

Worldwide, more than 600 million individuals are affected by allergic rhinitis (5). The burden of AR in India has been increasing steadily, with recent estimates suggesting that up to one-third of the population may be affected; recent studies have also reported incidence rates ranging from 20% to 30% (5–7). This growing prevalence highlights the need for effective and well-tolerated therapeutic strategies that can provide rapid and sustained symptom control in routine clinical practice (6, 7).

Intranasal corticosteroids (INCS) remain the cornerstone of pharmacological management for AR because of their potent anti-inflammatory effects targeting multiple components of the allergic cascade (8, 9). However, monotherapy with INCS may not adequately control symptoms in all patients, particularly those with moderate-to-severe disease or those experiencing both early-phase and late-phase allergic responses (10, 11). The Allergic Rhinitis and Its Impact on Asthma (ARIA) 2025 guideline recommends a combination of intranasal corticosteroid (INCS) and intranasal antihistamine (INAH) to be used as first-line treatment for allergic rhinitis because of its superior symptom control (12). The International Consensus Statement on Allergy and Rhinology 2023 consensus also highlights that intranasal corticosteroid and intranasal antihistamine combination therapy is more effective than monotherapy or placebo, offering rapid symptom control and improved overall efficacy, and is recommended when monotherapy fails to achieve adequate control (13). INCS–INAH FDC has been shown to provide greater reduction in total nasal symptom scores (TNSS) compared with corticosteroid monotherapy (14–19).

A multicentric, comparative study performed in 2020 found that both intranasal mometasone–azelastine and fluticasone furoate–azelastine combinations demonstrated comparable efficacy in relieving nasal and extranasal symptoms; however, a higher incidence of bitter taste and nasal irritation was reported among patients who received fluticasone furoate–azelastine (16).

Limited real-world data are available to evaluate the effectiveness and safety of the Mometasone furoate and Azelastine hydrochloride combination in patients with allergic rhinitis in India. Hence, this retrospective evaluation study was conducted to study the effectiveness and safety of Mometasone furoate and Azelastine hydrochloride NS in clinical practice for the management of patients with allergic rhinitis in the country.

## Methods

This was a multicentric, retrospective observational study that used the medical records of the patients prescribed Mometasone furoate and Azelastine hydrochloride intranasal spray by their treating physician. This study was conducted in accordance with the applicable regulations, good clinical practice (GCP), and Standard Operating Procedures. Ethics Committee (EC) approval was obtained before initiating the study (EC Approval Number: NIS/2024/14, Version No.: 1.0, dated 5-Sep-2024). Ethical approval was obtained on 5 September 2024 prior to data analysis. As this was a retrospective study using anonymized records, informed consent was waived by the ethics committee. Patient confidentiality was maintained through anonymized data capture.

Data were retrospectively collected for the period between January and August 2024. A total of 4,500 medical records from 424 ENT clinics were reviewed. Participating ENT clinics were included based on the availability of eligible patient records and participation in the electronic data capture system.

Patients with allergic rhinitis aged ≥12 years and prescribed with mometasone furoate and azelastine hydrochloride intranasal spray (Ryaltris AZ®) up to 14 days by the treating physician were included in the trial. Incomplete medical records/missing key data and the medical records of patients who received other types of AR treatment [oral antihistamines, leukotriene receptor antagonist (LTRA), etc.] were excluded if these medications were prescribed concurrently, other than the study drug.

A predesigned structured proforma was used for this study to collect information from sites retrospectively. Data were extracted by trained site personnel using a standardized structured proforma. Clinical data were collected by the clinical site personnel and entered into an electronic Case Report Form (eCRF). An eCRF designed on ClinSoft EDC was utilized for data collection. All information required by protocol was recorded onto the EDC system by designated investigator staff. Consistency was maintained through predefined data fields in the EDC system.

Patients were categorized based on the presence and impact of four key symptoms: sleep disturbance, impairment of daily activities, leisure, or sport, impairment of school or work, and the presence of troublesome symptoms. Mild allergic rhinitis was defined as the absence of all these symptoms. Moderate allergic rhinitis included the presence of one to three of these symptoms. Patients who experienced all four symptoms were classified as having severe allergic rhinitis. Symptom scoring was based on routine clinical documentation and may have varied across centers.

Descriptive statistics were used, and data were presented as absolute numbers/percentages. Changes in symptom scores from baseline to Days 7 and 14 were evaluated using paired *t*-tests. A two-sided *p*-value <0.05 was considered statistically significant. Statistical analyses were conducted using R software (version 4.5.3), a widely used platform for statistical computing and graphical analysis.

## Results

Among 4,500 screened records, 4,213 were included in the analysis; the mean (SD) age of the patients was 36.29 (±12.58) years. Male patients constituted 64.63%, whereas female patients constituted 35.37%; the mean duration of AR was 2.66 (±2.86) years. A summary of demographics and baseline characteristics is presented in Table 1.

**Table 1:** Summary of demographics and baseline characteristics.

Parameter	Category	Statistics (*N* = 4,213)
Age (years)	Mean (±SD)	36.29 (±12.58)
Gender *n* (%)	Male	2,723 (64.63%)
	Female	1,490 (35.37%)
Severity of allergic rhinitis (%) (*n* = 4,148)	Mild	993 (23.93%)
	Moderate	1,843 (44.43%)
	Severe	1,312 (31.62%)
Duration of allergic rhinitis (years)	Mean (±SD)	2.66 (±2.86)
Classification basis of the duration of allergic rhinitis *n* (%) (*N* = 4,114)	Intermittent	2,059 (50.04%)
	Persistent	2,055 (49.95%)
Nasal symptom score	Mean (±SD)	7.09 (±3.15)
Non-nasal symptom score	Mean (±SD)	5.25 (±3.56)

Percentages were calculated using the respective column header count as the denominator. SD, standard deviation; *N*, number of participants.

The most common presenting symptoms were cough and cold (reported in 14% of patients). The most common comorbid conditions with AR were diabetes mellitus (0.8%), hypertension (0.8%), asthma (0.5%), bronchitis (0.3%), and chronic rhinosinusitis (0.3%).

**Figure 1:**
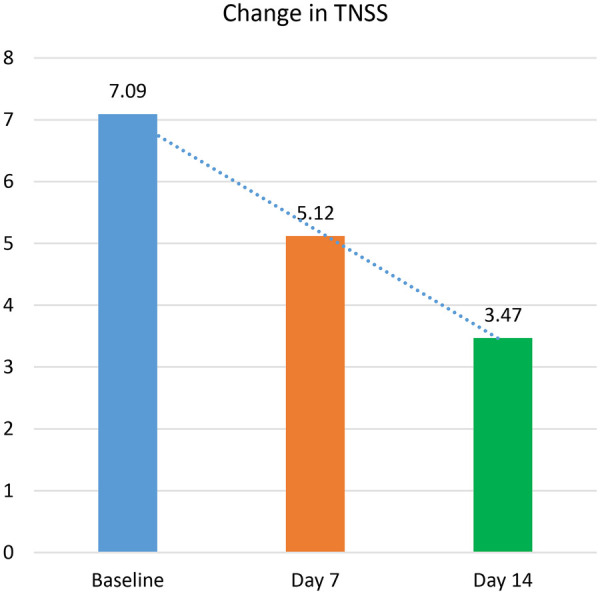
Mean change in total nasal symptom scores (sum of scores of nasal congestion, rhinorrhea, itching, and sneezing) from baseline to Days 7 and 14.

The mean change in total nasal symptom scores from baseline to end of treatment (14 days) is presented in Table 2 and Figure 1.

**Table 2:** Mean change in total nasal symptom scores (sum of scores of nasal congestion, rhinorrhea, itching, and sneezing) from baseline to Days 7 and 14.

Visit	Mean change (±SD)	*p*-Value
Mean change from baseline to Day 7 (*N* = 4,213)	−1.97 (±2.58)	*p* < 0.0001
Mean change from baseline to Day 14 (*N* = 4,213)	−3.62 (±3.99)	*p* < 0.0001

SD, standard deviation; *N*, number of participants. *p*-Values were calculated using a paired *t*-test.

A significant reduction was seen in the TNSS from baseline to Day 7 [−1.97 (±2.58, *p* < 0.001)] and Day 14 (−3.62 ± 3.99, *p* < 0.001).

The rate of change in the TNSS was 27.78% at Day 7 from baseline and 51.05% at Day 14 from baseline.

Significant reductions were seen in the nasal congestion score, rhinorrhea score, nasal itching score, and sneezing score at Day 7 from baseline and at Day 14 from baseline (Table 3).

**Table 3:** Mean change in individual nasal symptom scores.

Nasal symptom score	Mean change from baseline to Day 7	Mean change from baseline to Day 14
Nasal congestion score	−0.50 (±0.83)*	−0.93 (±1.16)*
Rhinorrhea score	−0.49 (±0.84)*	−0.92 (±1.14)*
Nasal itching score	−0.47 (±0.86)*	−0.85 (±1.13)*
Sneezing score	−0.51 (±0.94)*	−0.91 (±1.20)*

SD, standard deviation. *p*-Values were calculated using a paired *t*-test.

* *p* < 0.0001.

**Figure 2:**
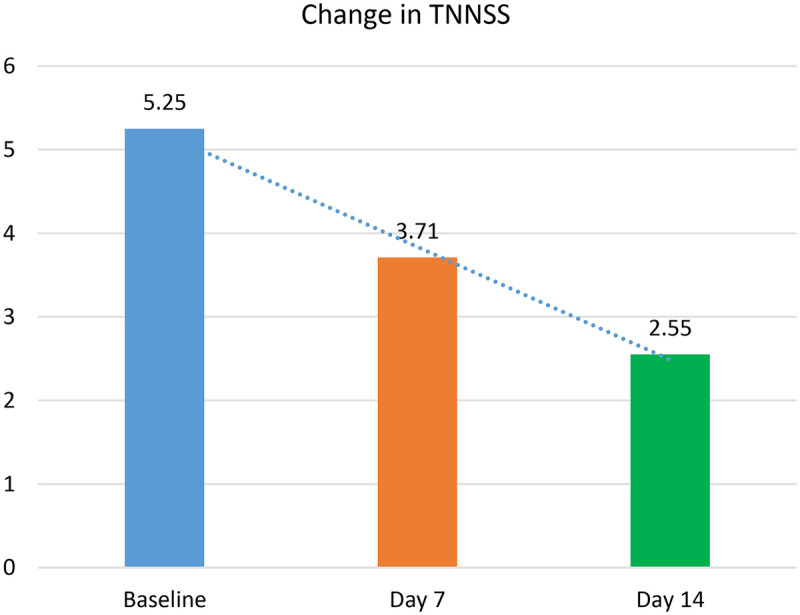
Mean change in total non-nasal symptoms scores from baseline to Days 7 and 14.

A significant reduction was seen in total non-nasal symptom scores from baseline to Day 7 (−1.55 ± 2.63, *p* < 0.0001) and Day 14 (−2.70 ± 3.84, *p* < 0.0001), as presented in Table 4 and Figure 2.

**Table 4:** Mean change in total non-nasal symptom scores from baseline to Days 7 and 14.

Visit	Mean change (±SD)	*p*-Value
Mean change from baseline to Day 7 (*N* = 4,213)	−1.55 (±2.63)	*p* < 0.0001
Mean change from baseline to Day 14 (*N* = 4,213)	−2.70 (±3.84)	*p* < 0.0001

SD, standard deviation, *N*, number of participants. *p*-Values were calculated using a paired *t*-test.

The rate of change in TNNSS was 28.65% at Day 7 from baseline and 51.42% at Day 14 from baseline.

Itching/burning eyes score, tearing/watering eyes score, redness of eyes score, and itching of ears or palate score also showed significant reductions at Day 7 from baseline and at Day 14 from baseline; these are represented in Table 5.

**Table 5:** Mean change in individual non-nasal symptom scores.

Non-nasal symptom score	Mean change from baseline to Day 7	Mean change from baseline to Day 14
Itching/burning eyes score	−0.41 (±0.84)*	−0.73 (±1.12)*
Tearing/watering eyes score	−0.40 (±0.84)*	−0.71 (±1.11)*
Redness of eyes score	−0.38 (±0.84)*	−0.66 (±1.09)*
Itching of ears or palate score	−0.36 (±0.86)*	−0.60 (±1.08)*

SD, standard deviation. *p*-Values were calculated using a paired *t*-test.

* *p* < 0.0001.

Adverse events (AEs): Out of the total study population, 28 (0.66%) patients experienced adverse events. No serious adverse events (SAEs) or discontinuations due to AEs were recorded during the study duration. No AE-related hospitalizations occurred. The number and summary of adverse events is presented in Table 6. Most AEs were mild and self-limiting. No SAEs or discontinuations were observed.

**Table 6:** Summary of adverse events.

Adverse events	*n* (%)
Allergy skin rashes	1 (0.02%)
Drowsiness	7 (0.16%)
Diarrhea	2 (0.04%)
Dryness, nausea	1 (0.02%)
Epistaxis	2 (0.04%)
Gastric irritation	1 (0.02%)
Hair loss	1 (0.02%)
Itching	3 (0.06%)
Mild irritation in the nostril	1 (0.02%)
Nasal swelling	1 (0.02%)
Redness	1 (0.02%)
Redness on the face	1 (0.02%)
Bitter taste	2 (0.04%)
Crusting in the nose	2 (0.04%)
Nasal irritation	1 (0.02%)

Percentages were calculated using the respective column header count as a denominator. *n* = No. of adverse events.

## Discussion

INCS is widely recommended as the cornerstone therapy for allergic rhinitis (AR) (10–12). Recent ARIA guidelines endorse INCS in combination with INAH for patients with moderate-to-severe disease, particularly as a step-up strategy where both early-phase and late-phase responses contribute to symptom burden (9). Fixed-dose INCS–INAH combinations have consistently demonstrated greater reductions in the total nasal symptom score (TNSS) compared with INCS alone, although the magnitude of the reductions may vary depending on corticosteroid pharmacodynamics and tolerability (15–19).

A meta-analysis by Soe et al. (21) identified mometasone as one of the most efficacious agents for reducing TNSS in moderate-to-severe AR. It has also shown consistent effectiveness in relieving nasal congestion, a key symptom often inadequately controlled with antihistamine monotherapy, and is associated with improved sleep and daily functioning (21). Its efficacy is further supported in conditions such as nasal polyposis (22) and asthma–AR overlap (23), highlighting its role as an important anti-inflammatory component in combination therapy.

Real-world evidence in India has shown that the MF–Az combination provides significant reductions in TNSS and TNNSS, with better tolerability than alternative combinations such as fluticasone–azelastine, particularly in terms of lower rates of bitterness and nasal irritation (25). Such tolerability advantages are clinically relevant in chronic conditions, where even mild adverse effects may impact adherence and overall treatment satisfaction.

Mometasone furoate also has a favorable pharmacokinetic profile, characterized by minimal systemic bioavailability, thereby reducing the risk of systemic corticosteroid-related adverse effects (24, 26).

In the present study, the MF–Az combination demonstrated a 51% reduction in TNSS at Day 14 along with a significant alleviation of both nasal and non-nasal symptoms and a low incidence of adverse events (0.66%), indicating a meaningful clinical benefit in a broad Indian AR population. These findings support its use in patients with moderate-to-severe and persistent symptoms requiring effective and well-tolerated therapy.

Overall, the evidence suggests that the MF–Az combination is a clinically relevant option within the INCS–INAH class, offering effective symptom control with a favorable tolerability profile in routine clinical practice (20).

However, the findings should be interpreted in light of the study limitations. This study is limited by its retrospective design and lack of a comparator arm, restricting causal inference and direct comparisons. The short follow-up period of 14 days captures only short-term outcomes. Variability in symptom scoring across centers and reliance on routine medical records may affect consistency. In addition, potential confounders such as prior and concomitant treatments were not controlled. Safety outcomes may be underestimated because of reliance on recorded adverse events rather than active surveillance. Despite these limitations, this study provides valuable real-world evidence from a large, multicentric Indian population, supporting the effectiveness and tolerability of the MF–Az combination in routine clinical practice.

## Conclusion

The study found that intranasal mometasone and azelastine spray was effective and well tolerated in relieving nasal and non-nasal symptoms. A significant reduction was observed by Day 7, with further reduction by Day 14 in Indian patients with allergic rhinitis. In addition, intranasal mometasone–azelastine spray was also found to be effective in alleviating nasal congestion, which is a particularly bothersome symptom for patients with allergic rhinitis.

## Data Availability

The raw data supporting the conclusions of this article will be made available by the authors without undue reservation.
